# Charing Cross Hospital

**Published:** 1852-07

**Authors:** 


### SURGERY.

### CHARING-CROSS HOSPITAL.

*Convulsive Movements of Stump; Removal of the Nervous Bulbs; Amputation at the Shoulder-joint; Persistence of the Spasmodic Jerking.* Under the care of Mr. HANCOCK.

When the considerable number of amputations which are performed in public and private practice is considered, it might in some degree appear surprising that so few cases of painful stump come before the profession. What, in fact, becomes of the divided extremities of the nerves, in the great majority of amputations? They must certainly experience some compression, by the formation of the cicatrix, and yet the greater number of stumps are painless, and very useful; whilst it is to that very compression and irritation that the rare cases of pain and convulsive movements are attributed. The more or less bulbous state of the extremity of the divided nerves has no doubt some influence on the development of unpleasat symptoms; but it must be recollected that nerves are almost always found bulbous in perfectly healthy stumps. Must we seek the explanation of the abnormal state of parts alluded to, in the patient's temperament; or in the peculiar manner in which the bulbs may be incorporated with the bone and muscles; or in the size of the bulbous extremities? It is perhaps to a combination of these three causes that the distressing symptoms which have been recorded may be ascribed; but it may be presumed that the first of these causes—viz: the patient's peculiarity of temperament—has the greatest influence on the phenomena, especially where, besides pain, convulsive movements of the stump are manifested.

Of the few patients who have suffered in the manner last mentioned, the larger number were women, and these almost always of a more or less hysterical temperament. But it is nevertheless true, that cases have been recorded, in which the pain in the stumps, and the spasms, excited hysterical attacks which had never been developed before.

This affection is so distressing, and so little under the control of remedies, that surgeons have been at great pains to ascertain the exact nature of the neuromata or nerve-bulbs which sometimes follow amputation, in the hope that that knowledge might lead the way to a method of avoiding their formation. It has been found that the bulbs are not composed of nervous filaments, but that the bulk is made up of exudation matter, distributed among the fibrillæ of the nerves; and there are few stumps which do not contain some of these bulbs. But it is probable, that when they increase beyond a certain size, and are compressed by a hard cicatrix, they excite the pain and convulsive movements which are sometimes noticed. If this be true, a second amputation offers a good chance of freeing the patient from these symptoms; but herein we do not always succeed, as will be seen by the case lately under Mr. Hancock's care.

Nor are there examples wanting to show that there may be, in certain subjects, such a confirmed propensity to this kind of neuritis, that the affection will spring up again and again, though the bulbous extremities may be removed, or even the whole stump taken off at the joint. In fact, pain and convulsive movements may occur without the formation of bulbs, the symptoms probably depending on the more or less nervous inflammation which attends the process of cicatrization. On this head Mr. Miller says, very justly: "In the case of neuralgia apparently dependent on neuromata, these may be taken away by incision, and the wound treated most carefully, yet the same painful feelings are very prone to return, before fresh neuromata have had time to form; and may continue, even when careful manipulation satisfies the surgeon that additional neuromata have actually not been produced." This will be very strikingly illustrated by the following case, which for several months excited a lively interest at the hospital. From notes taken by Mr. Dalton, the house-surgeon, we become acquainted with the following facts:—

Ann V——, aged twenty-nine, of fair complexion, blue and somewhat wild-looking eyes, was admitted April 10, 1851, under the care of Mr Hancock. She is evidently of a highly nervous and excitable temperament; has generally attended to household duties, without any fixed occupation, and seems to be affected with the peculiar hysterical tendency of exciting as much as possible the attention and sympathy of those with whom she comes in contact. The left arm has been amputated, at about the middle point between the head of the humerus and the condyles, and the stump is constantly and convulsively moving, principally in a direction from above downwards. The pectoral muscles, the trapezius, the scaleni, and the platysma myoides, are also strongly agitated, and the parts are never at rest except when the patient is asleep.

She states that she was seized, about sixteen years ago, with severe pain and inflammation of the elbow-joint, (without having met with any injury,) and soon after, a rather serious illness, probably continued or other fever. For these symptoms she was actively treated; but becoming worse, she repaired to town, and placed herself under the care of the late Mr. Morgan, at Guy's Hospital. Here the usual means employed for diseases of joints were carefully used, but to no purpose; and Mr. Morgan was at last obliged to take off the arm—a period of five years having elapsed between the first onset of the articular disease and this amputation. The patient returned to the country before the stump was completely cicatrized; the healing process was completed in about two months, and a firm cicatrix formed.

At the expiration of three months after the operation, and one after the entire closure of the wound, the stump began to move convulsively, most of the muscles of the shoulder and neck connected with the arm soon partaking of the spasmodic twitchings. This had been preceded for several days by very intense pain in the extremity of the stump. Soon afterwards, a collection of matter occurred in the part, as she was then several times seen by the late Mr. Aston Key, at St. Alban's, who recommended her to return to Guy's Hospital. She now stayed in that institution for several months, during which, blisters, setons, issues, ointments of various kinds, and general treatment, were tried with great perseverance, but without any relief to the convulsive action or to the pain. Among the means employed, was the tying down of the stump; but when the latter was well secured, and the movements completely prevented, the *whole body* used to be thrown into convulsions. The pain would sometimes radiate from the stump to the neck, back of the head, and down the spine, and convulsions occasionally were noticed in the left thigh, leg, and great toe. Even the right shoulder shook now and then violently, and became exquisitely painful. She often woke suddenly, and found both the left leg and the stump in violent convulsions, but the movements used mostly to cease during sleep. The patient, however, stated that she had often been told that the stump, even when she was sound asleep, would start occasionally. It was likewise noticed that, at the height of the convulsive action, the skin and cellular tissue of the cicatrix became violently retracted.

Some time after leaving Guy's, the patient was admitted into St. George's Hospital, where the series of remedies available and sometimes efficacious in such cases, were successively tried, but as none gave any relief, amputation of the shoulder joint was mentioned, and agreed upon, two years having elapsed since the removal of the arm. But at this time a new feature arose in the patient's case; severe epileptic fits set in, which occurred more or less frequently for the space of two years after she had left St. George's.

We now find that the girl fell into the hands of a mesmerist, and she states that the convulsive action generally stopped during the artificial sleep. But it is difficult to put any faith in the circumstances connected with this period of the patient's history, as everything coming from such quarters must be received with extreme caution. There can be no doubt, however, that the girl must have been made much worse by the mesmeric juggleries, and that her present nervous, hysterical, and excitable condition is principally owing to the constant irritation offered to her nervous system, during seven years mesmeric treatment. After this, the patient suffered so much pain and inconvenience, that she applied for admission at St. Bartholomew's Hospital, where she stayed about nine months. The best directed means were here again perfectly useless; the amputation at the shoulder-joint was proposed, but she seems to have been at that time unwilling to submit to this last resource. At St. Thomas's Hospital, whither the patient next repaired, Mr. Green and Mr. Travers were of opinion that there was no chance of any relief but by removal at the joint. Some incisions were here made into the stump, but she left without consenting to the operation proposed.

Frequent blisters were subsequently applied to the part, as well as to the spine; galvanism was tried, but all to no purpose. The violence of the fits would sometimes abate considerably, and then she improved in health. She has always exhibited signs of indolence, and within the twelvemonth which preceded her admission into Charing-cross Hospital she gained much flesh.

The patient has amused herself in casting up the number of some of the remedies which were used in the different institutions where she was admitted, and finds that the stump was lanced twenty-five times, that she had four hundred and ninety-five leeches, ninety-six blisters, &c. It should be particularly noticed that during all these years, the catamenia (which had begun at eleven) were always very regular.

Mr. Hancock endeavored at first to give tone to the system, and to overcome the hysterical and nervous state of the patient. For this purpose, the most approved means, as quinine, steel, zinc, morphia, &c. &c. were used for a considerable period, but without any very tangible effect. It was clear, after several months had elapsed, that a more direct interference would be required to subdue the convulsive action of the stump. There could be hardly any doubt but that the bulbs of the nerves were much enlarged, and that their great size would ever be in the way of a cure; Mr. Hancock therefore resolved, before having recourse to the complete removal of the stump, to dissect out the bulbous extremities of the nerves.

On the 22d of August, 1851, whilst the patient was under the influence of chloroform, Mr. Hancock made a longitudinal incision, from the shoulder downwards along the anterior aspect of the stump. When the parts were fairly laïd open, a bulbous mass, about the size of the last phalanx of the thumb, came into view. This was carefully freed from the surrounding parts, down to the bone, and removed. During the operation the brachial artery was divided, and immediately tied; the lips of the wound were brought together, and when the stump was placed upon the pillow, it was observed that the spasmodic movements had entirely ceased. This circumstance was extremely satisfactory, and confirmed the belief that the presence of the nervous bulbs had been mainly instrumental in the persistence of the symptoms through so many years.

This favorable state of things lasted about one month; the stump lay perfectly still, and hopes were entertained that this very obstinate affection was at last conquered; but the patient, having been seized by a fit of impatience, about four weeks after the last mentioned operation, the stump began again to be convulsed, and matters soon became as bad as ever. It was now evident that nothing short of amputation at the shoulder-joint would be of any avail. Mr. Hancock, therefore, proposed the operation to the girl, who readily consented. On the 12th of November, 1851, the patient having been narcotized with chloroform, Mr. Hancock performed amputation at the joint, making a good flap of the deltoid muscle. It was noticed that the stump, when severed from the body and placed on the table, continued to be convulsively agitated for several minutes. No pathological appearance of importance was revealed by the examination of the stump, except that the nervous trunks were closely adherent to the bone.

The chloroform affected the patient to a very great degree, for about twenty-four hours after the operation, the hysterical diathesis having a large share in the somewhat alarming symptoms which were observed. This doubtful state did not, however, last long; the patient progressed very favorably for several weeks, and it might now be fairly surmised that all the pain and inconvenienee which had been undergone for so many years, were arrested; but hardly had the cicatrix formed, about two months after the operation, when the muscular fibres forming the deltoid, those of the trapezius, the scaleni, a portion of the great pectoral, and even the platysma myoides, were again thrown into strong convulsive action. The movements were just as continuous and spasmodic as they had been in the stump. When the girl was not observed, the jerking was much less rapid than when she was either angry or excited. Some suspicion had previously existed, and was now made somewhat strong, that the movements were, perhaps, not completely involuntary, Mr. Hancock thought it now prudent to allow the girl to breathe the country air; she was therefore discharged, February 15th, 1852, about ten months after her admission.

This is certainly one of the worst cases of the kind which have as yet been put upon record, with the only exception that Mr. Hancock's patient had no aneurismal pulsations accompanying the convulsive action. This case fully confirms the very generally received opinion, that neuralgia and convulsive movement of stumps, depending on a bulbous state of the extremity of the divided nerves, are hardly amenable to treatment, and that even the very severest measures, such as a complete removal of the stump, are found inefficacious. That the hysterical diathesis so unmistakable in Mr. Hancock's patient, must have had a very large share in the production of the symptoms, cannot for a moment be doubted; but it is clear, on the other hand, that the extremities of the nerves were more bulbous than is the case in the generality of stumps, and that there was a material and tangible cause for the symptoms besides the highly nervous and hysterical temperament. Had the patient never been affected with the disease of the elbow joint, which led to the first amputation, it is very likely that the hysterical diathesis, which exercised its full force on the stump, would have manifested itself in one of the numerous ways belonging to hysteria.

We need not refer our readers to the paper of Mr. Langstaff, in the sixteenth volume of the *Medico-Chirurgical Transactions*, to show that the soundness of a stump depends very much on the manner in which the soft parts cicatrize, and that very severe symptoms are apt to ensue when the bulbs of the nerves, which form almost in every stump, are increased to such an extent as to assume a neuromatous character. As to the actual nature of the enlargement, a subject to which we alluded above, Mr. Langstaff says:—"I think it right to mention, that on cutting through these bulbous extremities of the nerves, there are no signs of enlargement of their natural structure, the thickening appearing to have been occasioned wholly by the deposition of lymph, the effect of inflammation in the cellular tissue covering the neurilemma."

Mr. Langstaff mentions, in the same paper, the case of a girl, twenty years of age, whose forearm had been taken off, and who, like Mr. Hancock's patient, suffered from pain and convulsive action in the stump, with the addition of aneurismal pulsation. Every kind of means was tried, at several hospitals, without any favorable results; at last Mr. Langstaff took off the arm above the elbow-joint; and, in describing the operation, he says:—"I drew out each nerve, to the extent of half an inch, from the surface of the stump, with a tenaculum, and cut through them to prevent their interrupting the progress of cicatrization, which I have done in other instances with success; and I was astonished that the division did not occasion greater pain than was complained of by the patient." "This patient was relieved of all the painful sensations she had so long been distressed with; she had no recurrence of hysteria, (which affection had been induced by the convulsions of the arm,) her health improved; a good stump was effected; and she is now (1830) able to obtain her livelihood." We have some reason to suspect that this same patient had, some time after the publication of the paper, a relapse; she then came under the care of Mr. Tyrrel, who performed amputation a little higher up, without effecting a cure. Mr. Bransby Cooper lastly removed the stump at the joint, and at this moment the soft parts about the shoulder are as convulsively agitated with this woman, as is the case with Mr. Hancock's patient.—*London Lancet.*

